# Combining sodium-glucose co-transporter-2 inhibitor with mesenchymal stem cells and brown adipose tissue (BAT) and white adipose tissue (WAT) transplantation to mitigate the progression of diabetic kidney disease: a pre-clinical approach

**DOI:** 10.1186/s13287-025-04358-7

**Published:** 2025-05-20

**Authors:** Stephany Beyerstedt, Marcella L. Franco, Alanah K. G. Carlos, Jaqueline Arjona, Gleice R. Josefi-Rocha, Bruno S. Barbosa, Maria Theresa A. Balby-Rocha, Andrei Furlan da Silva, Tuany Marques Reiter Alves, Melise Oliveira Mariano, Maria Clara Soares Klein, Érika Bevilaqua Rangel

**Affiliations:** 1 Albert Einstein Research and Education Institute, Hospital Israelita Albert Einstein, São Paulo, SP Brazil; 2 School of Medicine, Faculdade Israelita de Ciências da Saúde Albert Einstein, São Paulo, SP Brazil; 3 Nephrology Division, Federal University of São Paulo, Rua Comendador Elias Jafet, 755 – Morumbi, São Paulo, SP 05653-000 Brazil

**Keywords:** Mesenchymal stem cell, SGLT2i, Diabetic kidney disease, Podocyte

## Abstract

**Introduction:**

The increasing prevalence of Diabetes Mellitus (DM) correlates with a rising incidence of Diabetic Kidney Disease (DKD). DKD, a multifactorial condition, is characterized by activation of the renin–angiotensin–aldosterone system (RAAS), with angiotensin II playing a significant role in podocyte injury. While conventional treatments show potential in mitigating DKD progression, a combination of strategies is required to both impede its development and repair damaged structures.

**Methods:**

In this study, we explored the brown adipose tissue (BAT) and white adipose tissue (WAT) transplantation, and the use of bone marrow mesenchymal stem cell therapy (BM-MSC) combined with sodium-glucose co-transporter-2 (SGLT2) inhibitor treatment and calorie restriction in the BTBR^ob/ob^ model, recognized as a robust representation of DKD featuring hyperglycemia, obesity, time-dependent albuminuria, and histological changes.

**Results:**

Our primary findings revealed enhanced blood glucose control through combined cell therapy, diminished mesangial matrix expansion, alleviated tissue oxidative stress, preserved podocyte numbers, and an upregulation of podocyte structural markers and components of the RAAS renoprotective axis.

**Conclusion:**

BM-MSC therapy demonstrates considerable promise as a combined treatment for mitigating DKD progression, with similar findings observed for BAT and WAT transplantation.

**Supplementary Information:**

The online version contains supplementary material available at 10.1186/s13287-025-04358-7.

## Background

The global prevalence of Diabetes Mellitus (DM) is rising, with an estimated 537 million adults affected in 2019 and projections exceeding 783 million by 2045 [1]. This alarming increase is mirrored by a rise in its complications, particularly Diabetic Kidney Disease (DKD), which affects 30 to 40% of individuals with DM and is the leading cause of end-stage kidney disease (ESKD), posing a major public health challenge [2].

In DKD, dysregulation of metabolic pathways and the renin–angiotensin–aldosterone system (RAAS) synergistically contribute to podocyte injury. RAAS consists of two main axes: the ACE/Angiotensin II (Ang II)/AT1R axis, which causes inflammation, fibrosis, apoptosis, and oxidative stress; and the ACE2/Ang (1–7)/Mas axis, which has anti-inflammatory, antiapoptotic, and antifibrotic effects [3]. Chronic hyperglycemia drives aberrant intracellular glucose and lipid metabolism, generating reactive oxygen species (ROS) that induce oxidative stress. ROS primarily trigger tissue impairment by causing DNA damage, protein modification, lipid peroxidation, and disrupting cellular homeostasis, leading to apoptosis, senescence, reduced regenerative potential, and fibrosis [4].

In podocytes, hyperactivation of the RAAS further amplifies oxidative stress through Ang II-mediated activation of NADPH oxidase 4 and Transient Receptor Potential Channel 6 (TRPC6), resulting in increased cellular Ca^2+^ influx. This cascade culminates in podocyte cytoskeleton rearrangement and detachment, leading to increased glomerular permeability and albuminuria, hallmark features of DKD[5]. Therapeutic strategies targeting metabolic pathways, RAAS activation, and the restoration of renoprotective factors decreased in kidney diseases hold promise for mitigating podocyte injury and slowing disease progression [6].

Therefore, effective treatment requires a multifaced approach, including lifestyle changes, blood glucose control, and drug therapies, such as RAAS inhibitors and sodium-glucose co-transporter-2 (SGLT2) inhibitors. The latter is a recently added class that acts by inhibiting sodium and glucose reabsorption in the proximal tubule. This mechanism provides both hemodynamic effects, by reestablishing tubulo-glomerular feedback and reducing hyperfiltration, and non-hemodynamic effects, by decreasing inflammation, oxidative stress, and fibrosis. Preclinical studies and clinical trials have demonstrated that SGLT2 inhibitors are associated with lower rates of decline in estimated glomerular filtration rate (eGFR), reduced proteinuria, and consequently, a slowing in DKD progression [7, 8].

However, the current standard of care for DKD does not promote the regeneration of damaged structures. Given this limitation, mesenchymal stem cell (MSC) therapy presents a significant opportunity to bridge this critical gap. MSCs exhibit key properties for regenerative medicine, including a tropism towards damaged tissues and a rich secretome—a collection of secreted bioactive molecules with potent anti-inflammatory and pro-angiogenic properties [6, 9]. These characteristics, coupled with recent studies highlighting their renoprotective potential through the reduction of oxidative stress and preservation of podocytes, render them particularly promising candidates for therapeutic applications in DKD.

Another promising therapeutic approach for reducing the burden of DM and obesity involves increasing brown adipose tissue (BAT), a key regulator of thermogenesis, through the use of browning agents, which promotes the conversion of white adipose tissue (WAT) into BAT, and nanomedicine-based strategies [10]. Additionally, leptin, a hormone which is primarily secreted by WAT, plays a central role in neuroendocrine function, energy homeostasis, and metabolism, including the regulation of appetite and blood glucose [11–13].

In this study, our aim was to assess the therapeutic potential of combining empagliflozin, a SGLT2 inhibitor, with calorie restriction and bone marrow (BM)-MSC therapy compared to BAT and WAT transplantation in a murine pre-clinical model of type 2 DM (T2DM), obesity, and DKD.

## Material and methods

### Bone marrow mesenchymal stem cells (BM-MSC) characterization

We obtained and characterized the BM-MSCs as previously described in passages 7–10^(10)^. Briefly, these cells were acquired from male 6–8-week-old mT/mG mice (#007576; JAX Laboratories, Bar Harbor, ME, USA) through femoral/tibial bone marrow flushes, followed by purification in culture. Subsequently, BM-MSCs were identified by their fibroblast-like morphology and adherence to plastic in standard culture conditions. They were also characterized by the expression of surface molecules CD29, CD44, C90, CD105, and SCA-1, and the absence of CD34 and C45 (hematopoietic stem cell markers) and CD31 (endothelial cell marker) via flow cytometry immunophenotyping and differentiation assays (chondrogenic, adipogenic, and osteogenic lineages) [14].

### BTBR^ob/ob^

Experiments were performed in accordance with the Institutional Animal Care and Use Committees of Hospital Israelita Albert Einstein (HIAE), and the study was registered at the Jewish Institute of Research and Education, São Paulo, SP, Brazil (N°. 4450–20).

BTBR^ob/ob^ mice (BTBR.Cg-Lepob/WiscJ; 004824-JAX Laboratories, Bar Harbor, ME, USA), homozygous for leptin gene knockout, serve as a model for T2DM and DKD. In males, this model is characterized by hyperphagia, obesity, insulin resistance, and hyperglycemia by the 6th week of age, followed by the establishment of DKD with time-dependent proteinuria by the 8th week of age and histological changes, such as mesangial expansion and podocyte loss, resembling human DKD alterations [15].

Each experimental group contained six animals, and the mice were housed in individual cages with water ad libitum*.* They were maintained in a temperature-controlled environment on a 12-h light/dark cycle.

### Experimental design

In addition to the BTBR^ob/ob^ group, the following groups were studied:

#### SGLT2 inhibitor (SGLT2i) group

BTBR^ob/ob^ mice received empagliflozin formulated in chow ad libitum at 25 mg/kg (Jardiance®, Boehringer Ingelheim, Ingelheim am Rhein, RP, Germany).

#### SGLT2i + calorie restriction (CR) group

Calorie restriction (CR) was used as a complementary strategy to empagliflozin treatment. The CR intervention aimed to mimic changes in eating habits observed in clinical practice and has been shown to reduce DKD progression in BTBR^ob/ob^ [16]. Therefore, BTBR^ob/ob^ mice received empagliflozin-formulated chow with a calorie restriction calculated based on the chow consumption of the SGLT2i group without restriction. A CR of 30% was implemented, meaning CR mice received 70% of the amount of food consumed by the animals in the empagliflozin group.

Additionally, this group served as a control for the subsequent group injected with cell therapy. Therefore, the SGLT2i + CR mice received two doses of intraperitoneal injection with 500 µL of warm 0.9% sodium chloride solution (vehicle) at the 8th and 10th weeks of age.

#### SGLT2i + CR + BM-MSC group

BTBR^ob/ob^ mice were treated with empagliflozin-formulated chow in CR and administered BM-MSCs. The mice received BM-MSCs injections (in passages 10–11), containing 1.2 × 10^6^ cells each in warm 0.9% sodium chloride solution, intraperitoneally at the 8th and 10th weeks of age. Rapidly thawed BM-MSC vials were evaluated for cell viability by trypan blue staining and accepted if viability was greater than 80%.

#### BAT and WAT transplantation (Tx) group

One characteristic of BTBR^ob/ob^ mice is the reversibility of structural and functional manifestations of DKD with leptin replacement [17, 18].

To reverse DKD, we established a protocol involving the transplantation (Tx) of adipose tissue from BTBR wild-type and heterozygous mice to BTBR^ob/ob^. The methodology involves a pool of 8 to 12 donors, previously confirmed by genotyping (Figures S1 and S2A-C). Donors were euthanized by an overdose of 2.5–5% isoflurane (Cristália, Itapira, SP, Brazil), followed by cervical dislocation, and used for a single recipient. Inguinal white adipose tissue (WAT) and interscapular brown adipose tissue (BAT) were removed to achieve a final fat volume of 12–15% relative to the donor's weight. Once the required amount was reached, verified on a semi-analytical scale, the fat was cleaned to remove any remaining hairs, homogenized once, passed through a 3 mL syringe with a 16-gauge needle, and kept in cold ischemia.

For the recipient, a 4 to 5-week-old BTBR^ob/ob^ mouse was used. The animal was ventilated via a facial mask and anesthetized with an initial dose of 5% isoflurane, later maintained during surgery with a 2–3% dose of the inhalation anesthetic. Asepsis was performed with 2% chlorhexidine (Riohex®, Rioquimica, São José do Rio Preto, SP, Brazil) on the dorsal area of the animal, positioned in ventral decubitus. Subsequently, 100–200 µL of each type of adipose tissue was injected in regions near the collection sites. BAT was applied in the anterior region of the animal's back, near the interscapular region, while WAT was injected in the posterior region. At the end of the surgery, the animal was removed from the facial mask and received analgesia with tramadol hydrochloride (Cristália, Itapira, SP, Brazil) at a dose of 40 mg/kg intramuscularly.

### Functional assessment

Body weight was recorded weekly, and fasting blood glucose was measured biweekly from baseline. Plasma and urine samples were collected at baseline and during weeks 8, 10, 14–15, and 18–20, using a metabolic cage for urine collection. Biochemical analyses of creatinine, glycosuria, and natriuresis were conducted using a Cobas Mira Plus (Roche, Basel, Northwestern Switzerland, Switzerland). Additionally, albumin levels were assessed by ELISA (Mouse Albumin ELISA Kit; ab207620 and #ab108792, Abcam, Cambridge, MA, USA).

### Morphological analysis

Kidney sections (paraffin-fixed, 3.5 µm thick) were stained with hematoxylin–eosin (HE) to evaluate the glomerular area in 30 glomeruli per animal under light microscopy (magnification 40x). Pancreatic sections (paraffin-fixed, 3.5 µm thick) were also stained with HE to assess the area and integrity of pancreatic islets in up to 30 islets per animal using light microscopy (magnification 10x). Both measurements were performed using CellSens software (Olympus, Shinjuku, Tokyo, Japan).

### Mesangial expansion

Kidney Sects. (3.5 µm thick, paraffin-fixed) were stained with periodic acid-Schiff (PAS) trichrome. The increase in mesangial matrix was determined by the presence of PAS-positive area in the mesangium, measured in 30 glomeruli per animal under light microscopy (magnification 40x) using CellSens software (Olympus, Shinjuku, Tokyo, Japan).

### Immunohistochemistry (IHC) analysis

Immunohistochemistry was performed on pancreatic Sects. (5 µm thick, paraffin-fixed) to evaluate insulin staining and cleaved caspase-3 for apoptosis. In kidney Sects. (3.5 µm thick, paraffin-fixed), we assessed podocyte numbers and oxidative stress. The EnVision FLEX High pH kit (#K8000, DAKO, Carpinteria, CA, USA) was used for all reactions.

For insulin, a rabbit polyclonal insulin antibody (Insulin H-86; Sc-9168, Santa Cruz, Dallas, TX, USA) was applied, insulin positive areas were scored by counting the number of positive staining areas in pancreatic islets under light microscopy (magnification 20x) using CellSens software (Olympus, Shinjuku, Tokyo, Japan).

For cleaved caspase-3, a rabbit monoclonal cleaved caspase-3 antibody (Cleaved Caspase-3 -D175—5A1E, 9664S, Cell Signaling Technology, Danvers, MA, USA) was applied, cleaved caspase-3 positive areas were scored by counting the number of positive staining area in pancreatic islets under light microscopy (magnification 20x) using CellSens software (Olympus, Shinjuku, Tokyo, Japan).

At 14–15 weeks, a mean of 16 islet cells were counted per animal, while at 18–20 weeks, a mean of 9 islet cells were counted.

To assess podocyte numbers, a rabbit polyclonal WT-1 antibody (anti-WT1; #sc-192, Santa Cruz, Dallas, TX, USA) was applied. WT-1 + cells were counted in 30 glomeruli randomly chosen under light microscopy (magnification 40x) using CellSens software (Olympus, Shinjuku, Tokyo, Japan).

Oxidative stress was evaluated by staining for 4-HNE (anti-4-hydroxynonenal polyclonal antibody; #ab46545, Abcam, Cambridge, MA, USA). This parameter was analyzed in six sections of cortical and medullary sections per animal under light microscopy (20 × magnification) using CellSens software (Olympus, Shinjuku, Tokyo, Japan).

### RNA Extraction, cDNA synthesis and RT-qPCR assays

RNA was extracted from kidneys using the RNeasy mini kit (#74,106, QIAGEN, Hilden, NRW, Germany), quantified (10 ng/µL; NanoDrop, Thermo Fisher Scientific, Waltham, MA, USA), and used for cDNA synthesis with the High-Capacity cDNA Reverse Transcription Kit (#4,368,814, Applied Biosystems, Waltham, MA, USA).

qPCR reactions were conducted on the Thermocycler qPCR-QuantStudio 6 Flex System using the TaqMan Real-Time PCR Master Mix kit (Thermo Fisher Scientific, Waltham, MA, USA) and the following probes: nephrin (Nphs1: Mm01176615_g1); α-actinin-4 (Actn4: Mm00502489_m1); synaptopodin (Synpo2: Mm03809162_m1); NAPH oxidase 4 (Nox4: Mm01317080_m1); TRPC6 (Trpc6: Mm01176083_m1); ACE2 (Ace2: Mm01159003_m1—Ace2); ACE (Ace: Mm00802048_m1); WT-1 (WT1: Mm01337048_m1). GUSB (Mm01197698_m1) was chosen as the endogenous housekeeping gene. The 2–ΔΔCt method (fold change) was used to determine the relative expression values, normalized to BTBR wild-type mice.

### Statistical analysis

Results were presented as mean ± standard deviation or standard error of the mean. Statistical analysis began with the Shapiro–Wilk normality test. If the data distribution was normal, a one-way ANOVA (Analysis Of Variance) followed by Tukey's post-test was used for single-variable analysis. For repeated measurements over time, a two-way ANOVA or mixed model test was performed, accounting for differences in the number of observations in repeated measurements, with Geisser Greenhouse’s epsilon correction was applied for non-sphericity when necessary. If the sample means did not follow a normal distribution, the Kruskall-Wallis test or multiple tests with Sidak's correction for multiple comparisons were applied. Statistical analysis was performed using the GraphPad Prism 8 software (GraphPad Software, La Jolla, CA, USA), with p-values < 0.05 considered statistically significant.

## Results

### Metabolic and kidney functional parameters in the experimental groups

**Fig. 1:**
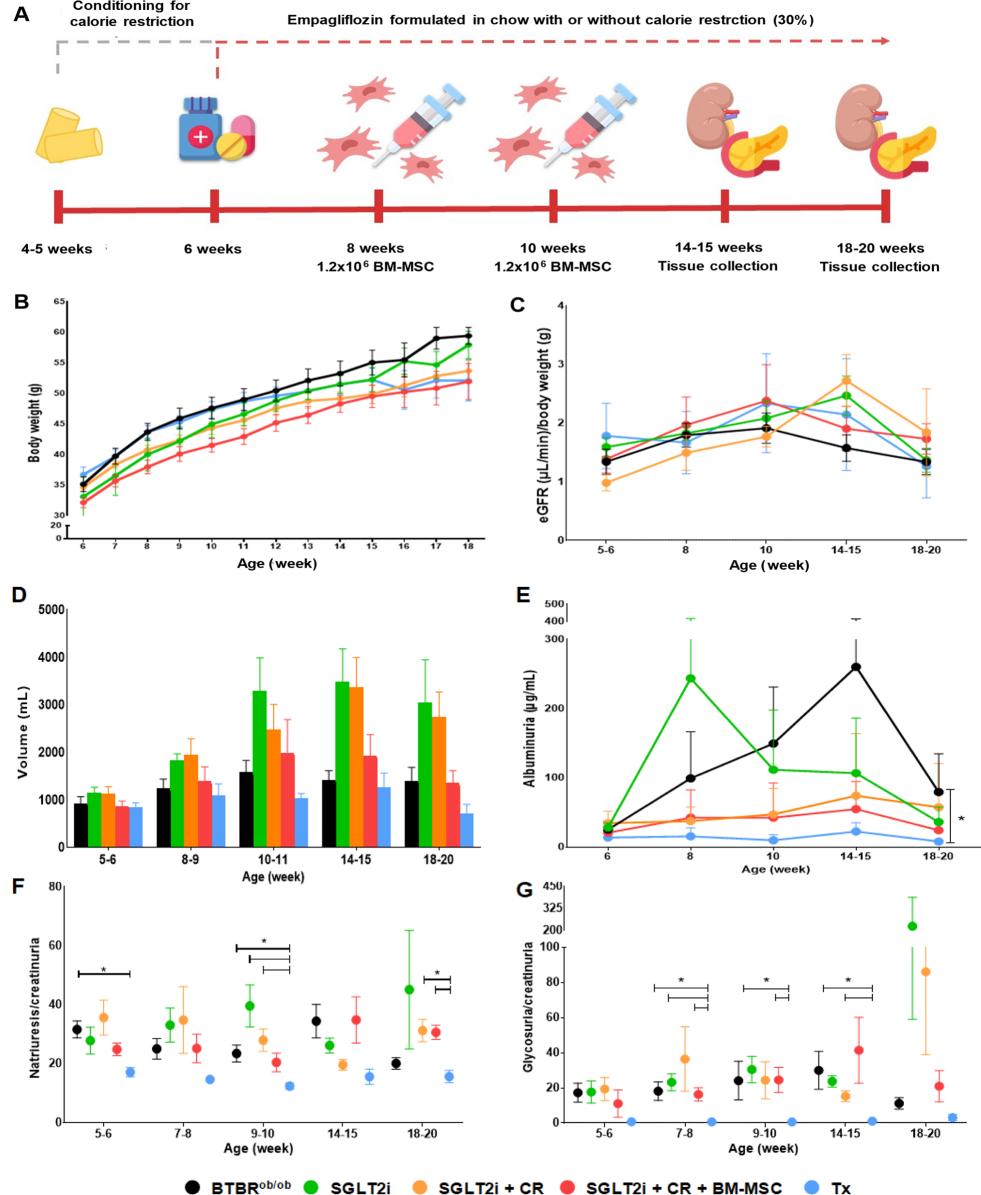
Metabolic and renal functional parameters in the experimental groups. **A** Schematization of the treatment protocols. Conditioning of the groups receiving calorie restriction and initiation of empagliflozin in the chow for SGLT2i-tretaed gropus occurred at 4–5 weeks of age. In the groups receiving combined therapy with BM-MSC, injections were administered at the 8th and 10th weeks of age. Euthanasia was performed at 14–15 and 18–20 weeks of age. **B** Body weight variation. **C** Estimated glomerular filtration rate (eGFR) normalized by body weight. **D** Urinary volume in mL. **E** Albumin measured in μg/mL over time. **F** Natriuresis normalized by creatinuria. **G** Glycosuria normalized by creatinuria. (*p < 0.05). Error bars represent mean ± SEM; n = 6–12 animals

As depicted in Fig. 1A, we implemented various experimental protocols over time. Notably, there were no significant changes in body weight between the groups, indicating that these strategies had no impact on obesity (Fig. 1B). Furthermore, we did not detect any effects on eGFR in our treatment groups compared to BTBR^ob/ob^, indicating no influence on hyperfiltration (Fig. 1C).

Drug therapy with SGLT2 inhibitors increased urinary volume through sodium excretion. In this context, we observed an increase in volume in the treatment groups, although not statistically significant (Fig. 1D).

In evaluating albuminuria, we observed a time-dependent effect in BTBR^ob/ob^, as anticipated. Our BAT and WAT transplantation (Tx) methodology significantly reduced albuminuria levels compared to the BTBR^ob/ob^ group, while the other treatment approaches did not achieve this result, despite showing potential for ameliorating albuminuria levels (Fig. 1E).

Additionally, we assessed natriuresis by normalizing it to creatinuria and detected a reduction in sodium excretion only in the Tx group compared to the other experimental conditions at specific time points (Fig. 1F).

In the glycosuria analysis, we did not observe significant changes in the therapy groups compared to the BTBR^ob/ob^ group, with reduced glycosuria levels detected only in Tx mice, as expected (Fig. 1G). We also evaluated fasting blood glucose to assess the impact of our approaches on DM (Table 1). The Tx group exhibited reduced blood glucose levels over time compared to the BTBR^ob/ob^. At baseline, lower blood glucose values were noted in the SGT2i + CR + BM-MSC group compared to the BTBR^ob/ob^ and SGLT2i groups, possibly due to conditioning with a reduced quantity of chow before exposure to SGLT2i.

**Table 1:** Variation in fasting capillary blood glucose over time in experimental groups

Age (weeks)	BTBR^ob/ob^	SGLT2i	SGLT2i + CR	SGLT2i + CR + BM-MSCs	BAT and WAT Tx
5–6	261(± 103.5)	253.4 (± 99.1)	164.3 (± 38.9)	144.6 (± 29)^a,b^	180.8 (± 64.3)
7–8	347.9 (± 100.7)	254.3 (± 76.1)	243.4 (± 44.1)	245.9 (± 55.1)^a^	193.1 (± 43.8)^a^
9–10	393 (± 113.9)	282 (± 79.3)	282.8 (± 82.7)	289.1 (± 87.9)	189.8 (± 52.7)^a,b,c,d^
11–12	377 (± 108)	275.6 (± 114.8)	270.6 (± 83.9)	230.5 (± 80.1)^a^	212.3 (± 41.3)^a^
14–15	464.8 (± 103.8)	343.3 (± 71.9)^a^	277.8 (± 81.9)^a^	203.8 (± 76.7)^a,b^	187 (± 59.9)^a,b^
16–17	361.5 (± 76.2)	288.8 (± 84.8)	285.8 (± 124.8)	219.2 (± 76.6)	198.8 (± 88)^a^
18–20	322.8 (± 72.5)	287.8 (± 94.9)	300.6 (± 85.2)	266.2 (± 115.2)	192.8 (± 31.7)^a^

^a^ *p* < 0.05 *vs* BTBR^ob/ob^, ^b^ *p* < 0.05 *vs* SGLT2i, ^c^ *p* < 0.05 *vs* SGLT2i + CR, ^d^ *p* < 0.05 *vs* SGLT2i + CR + BM-MSCs; mean ± standard deviation. Mixed effect model, with Tukey post-test for multiple comparisons. n = 6–12 animals

At 11–12 weeks of age, therapy with BM-MSCs demonstrated effectiveness, as blood glucose reduced in relation to BTBR^ob/ob^ mice, while the other interventions did not have this effect. However, at 14 to 15 weeks of age, the period during which we observed peak glycemia in BTBR^ob/ob^ mice, all therapeutic approaches significantly reduced hyperglycemia: SGLT2i; SGT2i + CR, SGT2i + CR + BM-MSCs, and Tx. Moreover, fasting blood glucose decreased in the SGT2i + CR + BM-MSC and Tx groups compared to animals that received only SGLT2i. However, this effect on blood glucose of both conventional interventions and cell therapy was not sustained at later points of 16 to 20 weeks, during which only the Tx group maintained effectiveness in reducing blood glucose compared to BTBR^ob/ob^ mice.

### Pancreatic islet evaluation

**Fig. 2:**
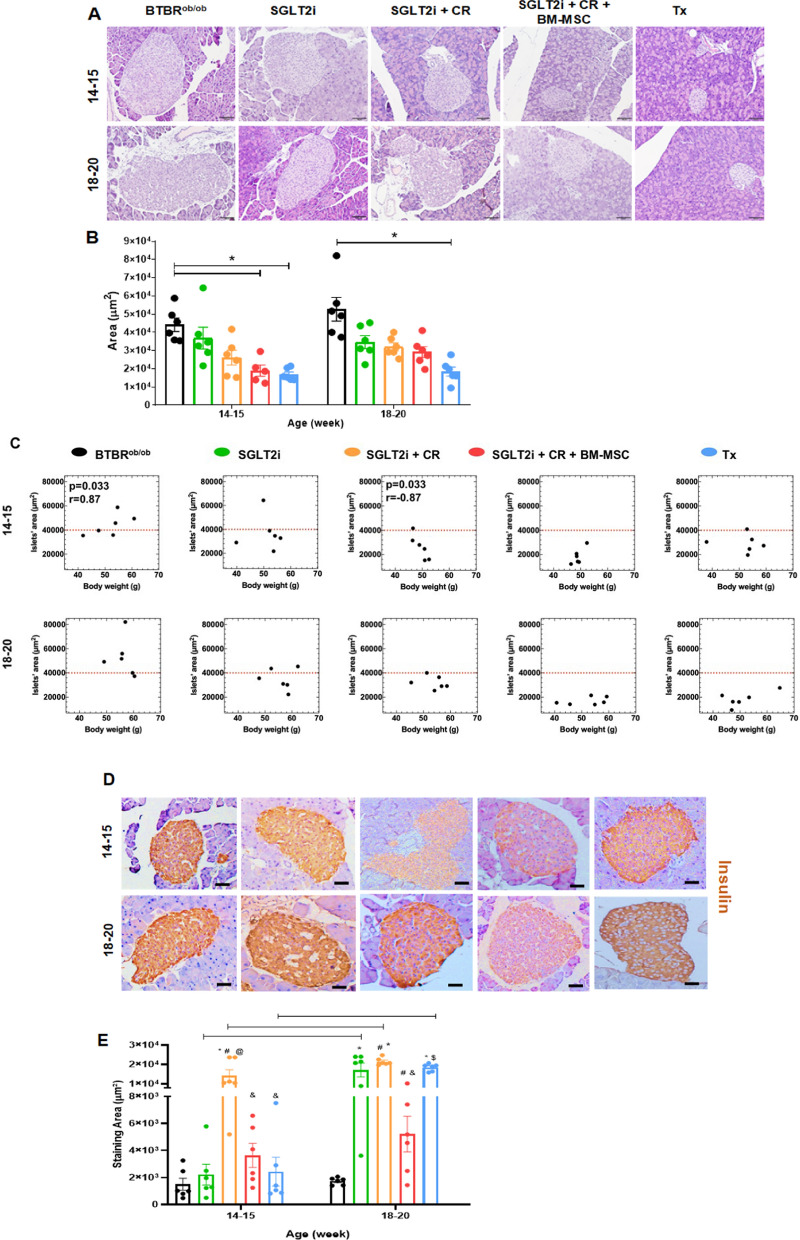
Pancreatic islet analysis. **A** Representative hematoxylin–eosin (HE) staining for morphological evaluation of pancreatic islets at 14–15 and 18–20 weeks of age. **B** Pancreatic islet area analysis across experimental groups. (* *p* < 0.05). **C** Spearman correlation between islets’ area (µm^2^) and body weight (g). **D** Representative images for insulin immunohistochemical analysis. **E** Quantification of insulin-positive cells per pancreatic islets across groups. (*p* < 0.05; * vs. BTBR^ob/ob^; # vs. SGLT2i; & vs. SGLT2i + CR; $ vs. SGLT2i + CR + BM-MSCs; continuous line indicated difference between time points). Scale bars represent 100 μm in **A** and **D**. Error bars represent mean ± SEM; n = 6 animals

BTBR^ob/ob^ mice exhibited hypertrophy of pancreatic islets. At 14–15 weeks of age, we observed that the combination of BM-MSCs with conventional treatment reduced islet volume compared to the BTBR^ob/ob^ group, a result similar to that seen in the Tx group (Fig. 2 A and B).

When we evaluated the correlation between body weight and islet area, we found a positive correlation in the BTBR^ob/ob^ group (r = 0.87, *p* = 0.03) at 14–15 weeks of age, indicating that as body weight increased, islet size also increased to compensate for the insulin resistance associated with higher body weight (Fig. 2C). Conversely, in the SGLT2i + CR group, this correlation was negative at the same time point, suggesting preservation of islet size despite weight gain (r = − 0.87, *p* = 0.033). In the other groups, we did not find a significant correlation at any time point, although animals treated with SGLT2i, SGLT2i + CR + BM-MSC, and BAT and WAT transplantation displayed similar biological characteristics to those of the SGLT2i + CR group and distinct from the BTBR^ob/ob^ group.

Next, we evaluated insulin staining in islet cells and found that the SGLT2i + CR group exhibited the largest staining area compared to the other groups at 14–15 weeks of age (Fig. 2D-E). Notably, at later time points (18–20 weeks), the BTBR^ob/ob^ group and the SGLT2i + CR + BM-MSC group presented lower levels of insulin, contrasting with the hyperinsulinism observed in the other groups. Across all groups, the insulin staining area in islet cells increased from 14–15 weeks to 18–20 weeks, except in the BTBR^ob/ob^ and SGLT2i + CR + BM-MSC groups.

**Fig. 3:**
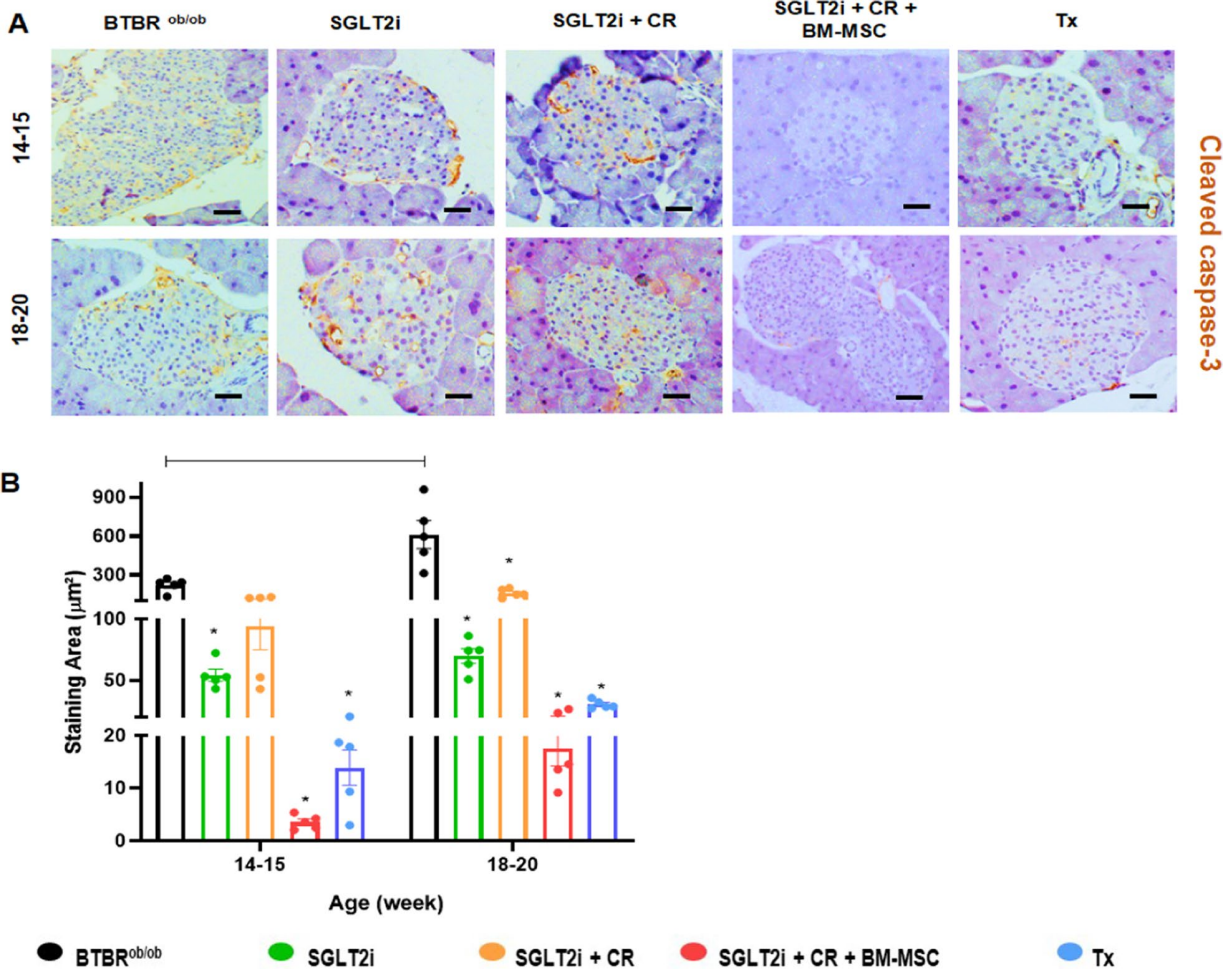
Immunohistochemical analysis of pancreatic islets. **A** Representative images of pancreatic sections from experimental groups stained for cleaved caspase-3 at 14–15 and 18–20 weeks of age. **B** Quantification of cleaved caspase-3-positive cells per pancreatic islets across groups. (*p* < 0.05; * vs. BTBR^ob/ob^; continuous line indicates difference between time points). Scale bars represent 100 μm in **A**. Error bars represent mean ± SEM; n = 5 animals

To further understand our findings on islet cell area and insulin staining, we investigated islet cell apoptosis using cleaved caspase-3 (Fig. 3A-B). We observed that the BTBR^ob/ob^ group and the SGLT2i + CR group exhibited the highest levels of apoptosis at 14–15 weeks of age. However, by the later time point of 18–20 weeks, only the BTBR^ob/ob^ group maintained high levels of cleaved caspase-3 staining, indicating that all treatments reduced islet cell apoptosis.

### Glomerular area and mesangial expansion evaluation

**Fig. 4:**
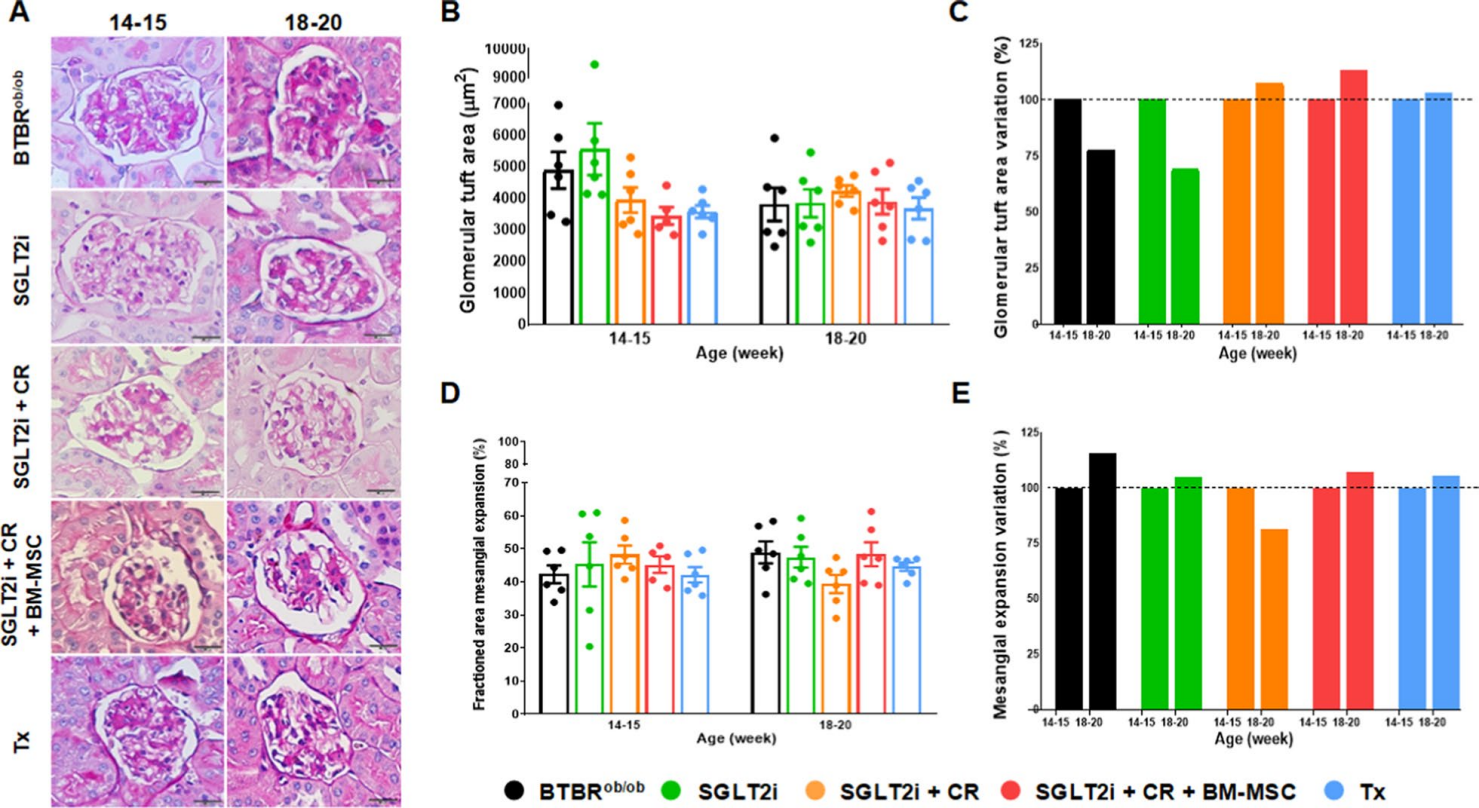
Analysis of glomerular hypertrophy and mesangial expansion. **A** Representative periodic acid-Schiff (PAS) staining illustrating the morphological evaluation of glomeruli at 14–15 and 18–20 weeks of age. **B** Evaluation of glomerular tuft area. **C** Progression of glomerular tuft area at 18–20 weeks of age compared to 14–15 weeks. **D** Fractioned mesangial expansion. **E** Progression of fractioned mesangial expansion at 18–20 weeks of age compared to 14–15 weeks. (*p < 0.05). Scale bars represent 20 µm in **A**. Error bars represent mean ± SEM; n = 5–6 animals

We assessed the impacts of the tested approaches on glomerular hypertrophy and mesangial matrix deposition **(**Fig. 4A). Regarding the glomerular area, no statistical differences were observed between the groups at either 14–15 or 18- 20 weeks of age (Fig. 4B). However, when we evaluated the progression of this parameter at the late DKD time-point compared to the values obtained at 14–15 weeks, we found that the BTBR^ob/ob^ and SGLT2i groups exhibited a reduction in glomerular area of approximately 23% and 31%, respectively. In contrast, the other groups showed increases, with increments of around 7.3% in the SGLT2i + CR mice, 12.9% in the SGLT2i + CR + BM-MSC, and 2.8% in the Tx group (Fig. 4C).

Regarding mesangial expansion, no significant differences were found between the experimental groups at either time point (Fig. 4D). However, when comparing the progression of this parameter from 14–15 weeks to 18–20 weeks, a greater increase was noted in the BTBR^ob/ob^ group (15.8%). In contrast, other animals showed an attenuation in mesangial expansion, with the SGLT2i, SGLT2i + CR + BM-MSC, and Tx groups displaying increases of 4.8%, 7.1%, and 5.8%, respectively, and an 18.5% reduction in the SGLT2i + CR group (Fig. 4E), consistent with histological findings (Fig. 4A).

### BM-MSC promoted podocyte number preservation

**Fig. 5:**
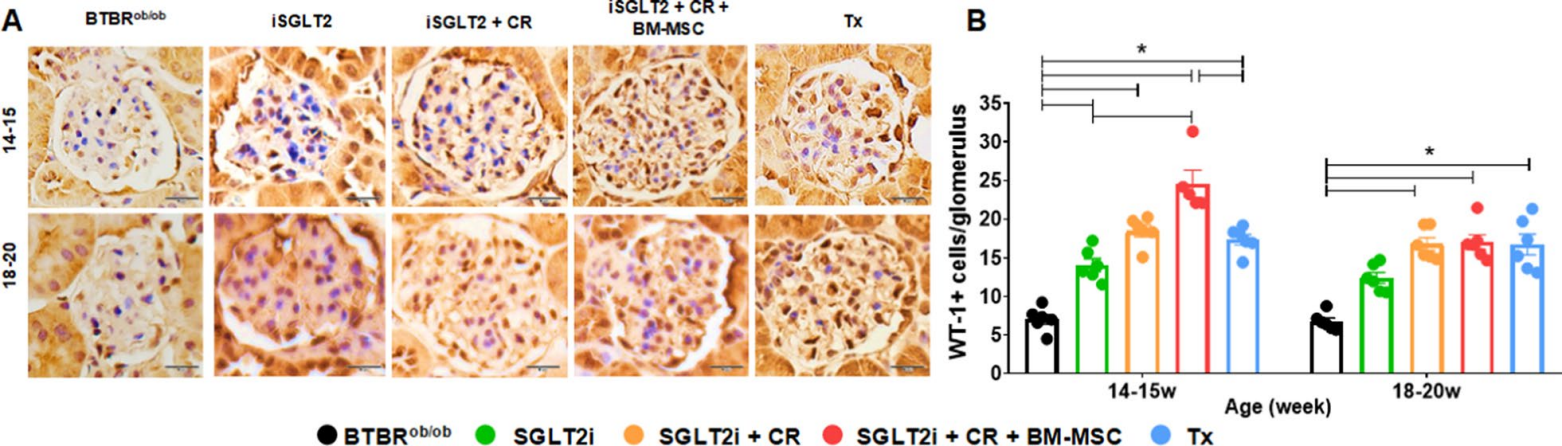
Immunohistochemical analysis of podocyte count. **A** Representative images of kidney sections from the experimental groups stained for the podocyte nuclear marker WT-1. **B** Evaluation of positive WT-1 cells per glomerulus among the different groups at both time points. (*p < 0.05). Scale bars represent 10 µm in **A**. Error bars represent mean ± SEM; n = 5–6 animals

We investigated the potential of our approaches to protect podocyte by quantifying positive cells for the podocyte nuclear marker WT-1 (Fig. 5A). As anticipated, the Tx group demonstrated preserved podocyte numbers, displaying a higher count of positive cells compared to the BTBR^ob/ob^ mice. Furthermore, our observations indicate that the combination of strategies with BM-MSC holds significant potential for podocyte protection, as the group treated with cell therapy exhibited an increased number of WT-1 + cells compared to both the BTBR^ob/ob^ and Tx groups at 14–15 weeks. Importantly, this protective effect persisted compared to the BTBR^ob/ob^ group until 18–20 weeks (Fig. 5B).

### SGLT2i and BM-MSC attenuated tissue oxidative stress

**Fig. 6:**
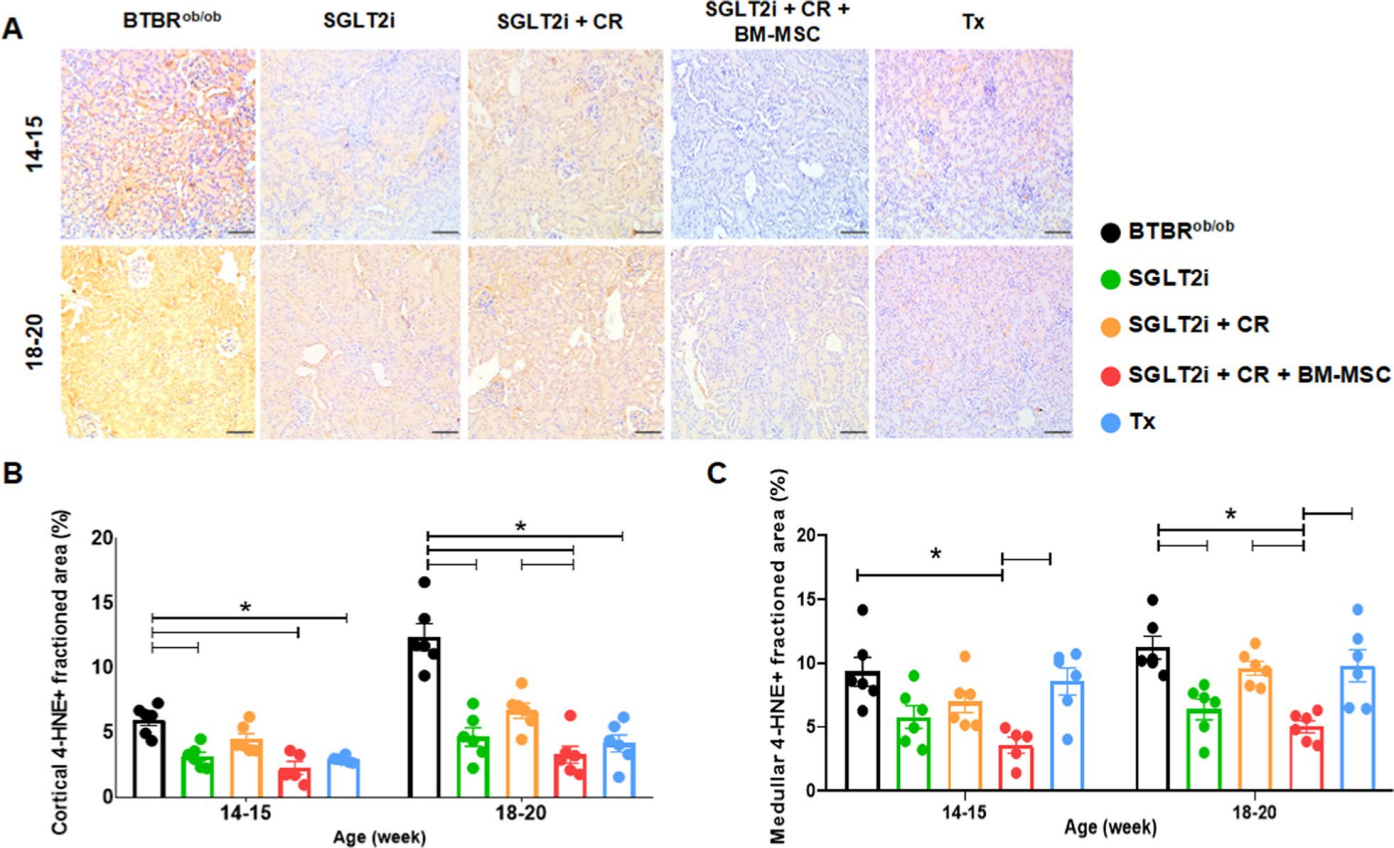
Immunohistochemical analysis of tissue oxidative stress via lipid peroxidation using 4-HNE. **A** Representative images of cortical sections from the experimental groups. **B** Evaluation of lipid peroxidation in the cortical region. **C** Evaluation of lipid peroxidation in the medullary region. (*p < 0.05). Scale bars represent 100 µm in **A**. Error bars represent mean ± SEM; n = 5–6 animals

Next, we examined the impact of our strategies on oxidative stress, a process associated with podocyte damage and the progression of DKD. Utilizing the lipid peroxidation marker 4-HNE (Fig. 6A), we observed a reduction in cortical staining in the SGLT2i, SGLT2i + CR + BM-MSC, and Tx groups compared to the BTBR^ob/ob^ at both time points (Fig. 6B). Furthermore, we observed the potential of these therapies to alleviate oxidative stress in medullary sections. There was reduced staining in the SGLT2i, SGLT2i + CR + BM-MSC, and Tx groups compared to BTBR^ob/ob^ group, with particularly pronounced reduction in the SGLT2i + CR + BM-MSC group compared to the SGLT2i + CR group (Fig. 6C).

### Gene expression of podocyte markers and RAAS components

**Fig. 7:**
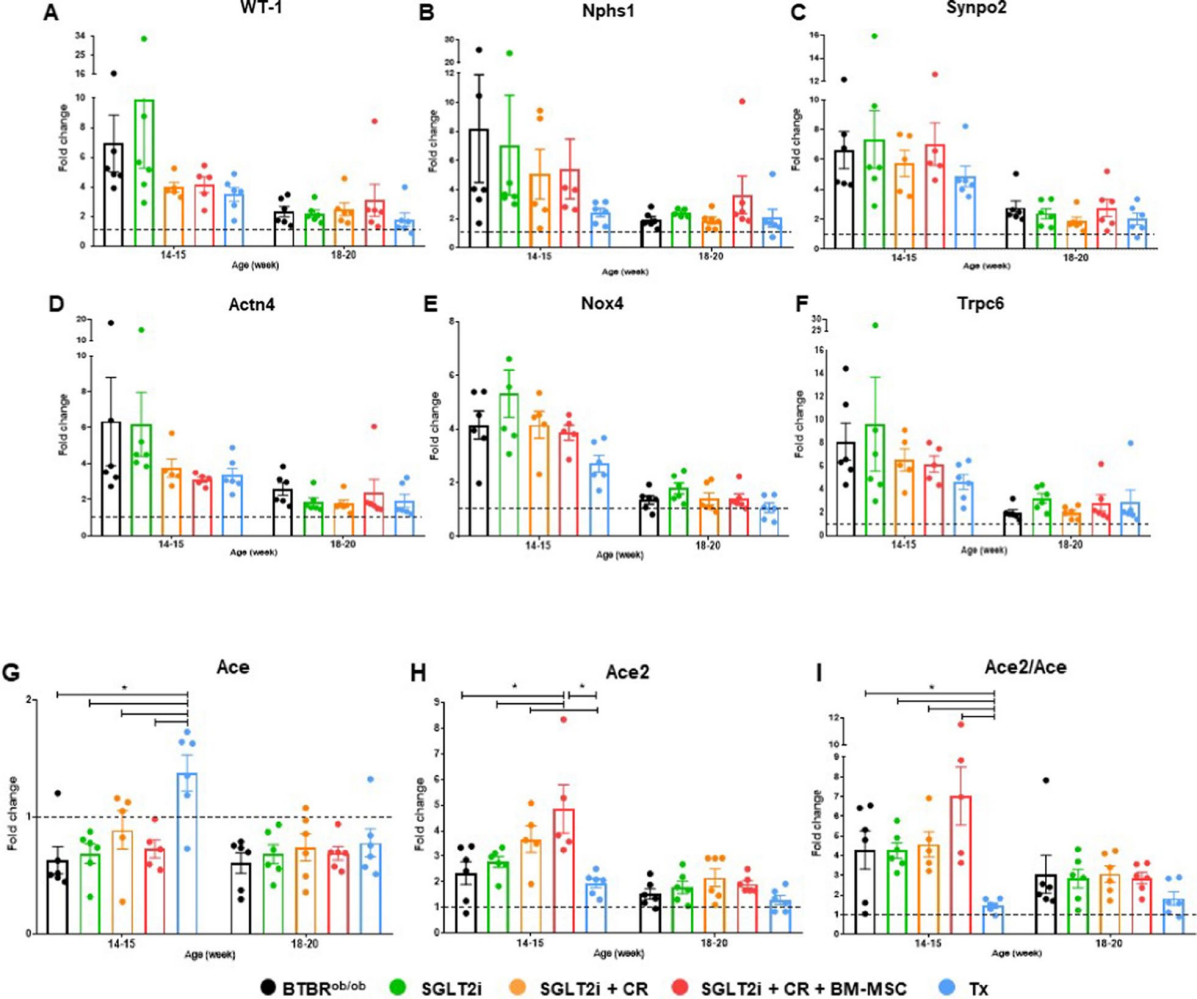
Gene expression of podocyte markers and components of the RAAS and Ang II/Nox4/TRPC6 axis compared to BTBR wild type. **A** WT-1. **B** Nphs1. **C** Synpo2. **D** Actn4. **E** Nox4. **F** Trpc6. **G** Ace. **H** Ace2. **I** Ace2/Ace ratio. (*p < 0.05). Error bars represent mean ± SEM; n = 5–6 animals

To assess the impact of our approaches on the expression of podocyte markers, we initially investigated the expression of WT-1 (Fig. 7A). All experimental conditions exhibited increased WT-1 gene expression compared to BTBR wild-type at both time points; however, we did not observe significance between the groups.

This trend of higher gene expression compared to the BTBR wild type was also evident in the analysis of other podocyte markers. For the nhps1 gene, encoding nephrin responsible for maintaining the structure and function of the diaphragmatic slit, we observed a higher potential of the combined therapy to upregulate this gene at 18–20 weeks of age (Fig. 7B). However, for genes encoding cytoskeleton proteins, synaptopodin (Fig. 7C), and α-actinin-4 (Fig. 7D), we were unable to identify significant differences between experimental groups.

Regarding the expression of components of the Ang II/Nox4/TRPC6 pathway, we observed higher expression of Nox4 (Fig. 7E) and Trpc6 (Fig. 7F) at both points compared to BTBR wild type, while no significance was identified among groups.

Furthermore, we investigated the expression of the ace and ace2 genes, which encode ACE and ACE2, respectively, to assess the activation of the intra-renal RAAS. We identified an increase in ACE in the Tx group, with significance observed between Tx and the other experimental groups (Fig. 7G). In ACE2 expression, all experimental groups exhibited increased levels compared to BTBR wild type. Notably, animals receiving SGLT2 intervention showed augmented ACE2 gene expression, with the combination with BM-MSCs proving more effective than monotherapy in targeting the ECA2/Ang (1–7)/Mas pathway at 14 to 15 weeks. This elevation persisted compared to BTBR^ob/ob^ animals until 18 to 20 weeks of age (Fig. 7H). Finally, the Ace2/Ace ratio indicated a potential targeting of the ACE2 renoprotective pathway (Fig. 7I).

## Discussion

This study aimed to assess the potential of MSCs to synergistically complement conventional treatments for DKD, thereby mitigating its progression. Our key findings highlight the ability of cell therapy to enhance blood glucose control, reduce the progression of histological changes in DKD, attenuate oxidative stress, and promote podocyte protection through modulation of RAAS activation.

Additionally, we introduced a novel method to reverse the diabetic phenotype in our model. While previous studies achieved reversion of DKD features through exogenous leptin administration using osmotic pumps [17, 18], our BAT and WAT transplantation methodology yielded similar results. These included diminished hyperglycemia, reduced glycosuria, attenuated mesangial expansion, and preserved podocyte numbers. Notably, our leptin replacement approach did not affect body weight, a parameter for which we did not observe significant changes in any of the treatment strategies. Consequently, there is potential for combining SGLT2i with other medications targeting satiety and body weight, such as GLP-1 (glucagon-like peptide-1) receptor agonists [19, 20].

Our study was designed with translational potential for clinical practice. We utilized a 25 mg dose of SGLT2i administered orally in the chow to mimic clinical settings. The choice of intraperitoneal MSC infusion, a safe and effective method allowing systemic distribution and kidney homing in renal damage scenarios, further aligns with clinical considerations [21].

MSCs from different sources, such as bone marrow (BM) and adipose tissue, possess distinct functional properties. While BM-MSCs have a greater capacity to differentiate into bone and fat cells compared to cartilage cells, their low in vivo differentiation efficacy emphasizes the importance of their secretoma [9]. In a pro-inflammatory microenvironment, typical in DKD, these cells acquire an immunosuppressive phenotype, secreting IL-10, prostaglandin E2, and indoleamine 2,3-dioxygenase [6, 22].

Given that hyperglycemia is the primary driver of DKD pathophysiology, our study extends previous findings indicating that MSC monotherapy does not effectively reduce blood glucose in the BTBR^ob/ob^ model [14]. Combining MSC therapy with the gold standard treatment revealed superior control of hyperglycemia, accompanied by attenuated pancreatic islet hypertrophy. This aligns with existing literature, supporting the theory that MSCs improve blood sugar levels by enhancing insulin sensitivity and improving pancreatic beta cell function, leading to increased insulin secretion. Additionally, MSCs modulate the expression and availability of glucose transporters in the plasma membrane, such as GLUT4 in skeletal muscle, adipose tissue, and liver, thereby reducing peripheral insulin resistance [23, 24].

Furthermore, we identified the time-dependent effect of cell therapy. We observed its ability to control blood glucose up to four to six weeks post-injection; however, this effect was not sustained at later time points. This observation aligns with increasing evidence of the short-term availability of MSCs in the organism after administration [25–27] and underscores the potential need for additional doses to achieve a long-term therapeutic effect [24, 28].

In the SGLT2i + CR group, a high insulin-staining area was observed despite elevated levels of apoptosis in islet cells. This finding may be attributed to the lower insulin resistance in this group, which could result in a higher rate of β-cell neogenesis and replication alongside cell death.

Conversely, in the BTBR^ob/ob^ group, islet cell hypertrophy was associated with increased body weight and hyperglycemia, which led to a reduced insulin-staining area due to sustained islet cell apoptosis and insulin resistance. This model, therefore, reflects the natural history of beta-cell adaptation in obesity and type 2 DM. Following an initial susceptibility phase influenced by genetic factors and postnatal nutrition, β-cells progress to an adaptive phase characterized by β-cell mass expansion and insulin secretion, accompanied by increasing weight and insulin resistance. As this scenario worsens, β-cell failure ensues due to glucolipotoxicity, oxidative stress, endoplasmic reticulum stress, and de-differentiation, ultimately resulting in elevated glucose levels and a decrease in β-cell mass [29]. Apoptosis is a primary mechanism of β-cell mass reduction [30], as demonstrated in our study. During the progression of diabetes, both intrinsic apoptosis, triggered by mitochondrial dysfunction, and extrinsic apoptosis, mediated by tumor necrosis factor-α (TNF-α), contribute to β-cell failure [31, 32]. Additional mechanisms involved in β-cell mass reduction include endoplasmic reticulum stress, oxidative stress, inflammation, and autophagy dysregulation.

Notably, in the SGLT2i + CR + BM-MSC group, no islet cell hypertrophy was observed, and insulin levels were maintained, likely due to reduced islet cell apoptosis and preserved β-cell mass. In the Tx group, islet cell hypertrophy was also absent, and insulin-staining area increased over time despite lower levels of islet cell apoptosis. It is plausible that the increased insulin-staining area reflects the release of insulin to control hyperglycemia in the setting of obesity and insulin resistance (Fig. 1B and Table 1), as this group was the only one to exhibit good glycemic control at later time points (18–20 weeks). BAT transplants may also contribute to insulin-independent reversal of DM by influencing WAT through several mechanisms, including the restoration of healthy functional WAT, an increase in M2 macrophages, and reductions in inflammation, lipolysis, and hypertrophy. These effects are accompanied by systemic benefits, such as decreased levels of the pro-inflammatory cytokines monocyte chemoattractant protein 1 and interleukin 6, increased thermogenesis, enhanced glucose uptake, elevated adiponectin levels, and higher insulin-like growth factor 1 (IGF-1). Additionally, BAT transplants have shown pancreatic effects, including decreased insulitis and reduced α-cell glucagon release [33].

Adipocyte secretome, particularly from BAT, acts on multiple target tissues by releasing endocrine factors that influence the liver, brain, pancreas, heart, skeletal muscles, bone, and WAT, with paracrine effects on the vascular and nervous systems and immune cells, as well as autocrine roles. In the context of DM and obesity, increasing BAT function or mass improves metabolic parameters and promotes weight reduction [10], along with functional and structural cardiac improvements [34]. Mechanistically, the transplantation of differentiated brown pre-adipocytes from BAT or those derived from human pluripotent stem cells has been shown to improve glucose homeostasis and insulin sensitivity through the AKT and FGF21 signaling pathways [35]. Furthermore, the metabolic benefits observed with BM-MSC treatment in our study may stem from these cells' potential to differentiate into both BAT and WAT [36]. Additionally, MSCs present within the adipose tissue could have contributed to paracrine effects. This has been documented elsewhere, highlighting their anti-apoptotic, antioxidant, and anti-inflammatory properties in diabetic animal models [9].

Regarding the kidney hallmarks of DKD, the combined strategy led to both the attenuation of mesangial matrix expansion progression and albuminuria, along with an increase in podocyte numbers compared to BTBR^ob/ob^. Glomerular mesangial cells play a crucial role in supporting glomerular structure and function by remodeling mesangial matrix. However, hyperglycemia leads to excessive matrix deposition and cellular exhaustion, ultimately exacerbating mesangial expansion. In pre-clinical models of DKD, MSC therapy has demonstrated efficacy in reducing mesangial matrix through the secretion of soluble factors, including vascular endothelial growth factor expression [37].

Moreover, the improvement in albuminuria over time can be attributed to an elevated podocyte count per glomerulus in the BM-MSC group. In a previous report, we highlighted the potential of MSC therapy to preserve podocyte numbers in this model [14]. Now, with combined strategies, we observe enhanced protection compared to conventional monotherapy approaches.

Podocyte injury in DKD is a multifactorial process, with oxidative stress emerging as a major contributor to damage in podocytes and other renal structures through lipid peroxidation, for example[38]. In this context, our study reveals that SGLT2i, both as a standalone intervention and in combination with cell therapy, significantly reduces lipid peroxidation in glomerular and medullary sections. Consequently, we identified podocyte protection as a potential outcome of attenuating tissue oxidative stress. These results are likely attributed to the modulation of RAAS activation by our strategies.

DM is associated with an imbalance in ACE/ACE2 activation, which leads to the activation of the ACE/Ang II/AT1R axis, causing exacerbated inflammation, fibrosis, apoptosis, and oxidative stress [3]. However, our study demonstrated the upregulation of the enzyme ACE2, indicating activation of the renoprotective ACE2/Ang (1–7)/Mas axis.

Additionally, SGLT2i exerts protective effects through direct action on podocytes. These cells express SGLT2, and its upregulation occurs in the context of advanced glycation end-products (AGEs)-induced glucotoxicity and oxidative stress [39]. Therefore, inhibiting SGLT2 prevents the loss of podocyte pedicels, detachment, and apoptosis by increasing autophagy, modulating energy metabolism, and decreasing Ang II action [7, 40].

Our study also revealed that BTBR^ob/ob^ mice exhibited elevated gene expression compared to wild-type mice. BTBR^ob/ob^ mice demonstrate hypertrophy with the induction of several genes related to the glomerulus and tubule, such as increased expression of E-cadherin and SGLT2 [18, 41]. Therefore, glomerular hypertrophy may, at least in part, explain the increased expression of podocyte markers.

Although promising, our study had several limitations. Firstly, as mentioned previously, we did not achieve body weight control with our approaches. Obesity is associated with hyperfiltration as a compensatory response to the increased metabolic demands linked to excess body weight [42], which may explain the lack of discernible effects on eGFR upon treatment. Furthermore, there were also limitations regarding the model. Oxidative stress contributes to disease progression by leading to tubulointerstitial fibrosis and glomerulosclerosis [4]. However, the BTBR^ob/ob^ model does not develop these characteristics robustly due to its insufficient life span for these features to become pronounced [15].

## Conclusions

In conclusion, our findings suggest that cell therapy can complement the gold- standard management of DKD (empagliflozin and calorie restriction) by improving blood glucose control, preventing islet cell hypertrophy and apoptosis, alleviating kidney oxidative stress, and enhancing g podocyte protection. Therefore, MSC therapy holds great promise as a combined treatment to mitigate DKD progression. Furthermore, BAT and WAT transplantation exhibited similar therapeutic properties when compared to MSC therapy combined to the gold-standard treatment to DKD, although achieving a more sustained reduction in albuminuria and improved glycemic control. These results highlight significant biological and therapeutic implications for DKD management.

## Supplementary Information

Additional file1 (DOCX 541 KB)

## Data Availability

The data that support the findings of this study are available on request from the corresponding author, É.B.R.
