# Supplementary material for: Combining sodium-glucose co-transporter-2 inhibitor with mesenchymal stem cells and brown adipose tissue (BAT) and white adipose tissue (WAT) transplantation to mitigate the progression of diabetic kidney disease: a pre-clinical approach
Source: Stem Cell Res Ther. 2025 May 20;16:254. doi: 10.1186/s13287-025-04358-7 (PMC12093872; doi:10.1186/s13287-025-04358-7)
Supplement: Supplementary file 1 — Additional file1 (DOCX 541 KB) [file 13287_2025_4358_MOESM1_ESM.docx]

**SUPPLEMENTARY MATERIAL**

**MATERIALS AND METHODS**

**Genotyping**

BTBR^ob/ob^ animals were phenotypically identified, and their genotype was confirmed through genotyping for the leptin gene allele. The genotyping process involved collecting a segment of the animal's tail, followed by DNA extraction using a lysis solution containing 25 mM NaOH and 0.2 mM EDTA. This extraction was carried out for one hour at 98°C with agitation at 300 RPM (rotations per minute). After incubation, the reaction was stopped by adding a 40 mM Tris-HCl pH 5.5 solution, followed by centrifugation at 10,000 RPM at 4°C for 10 minutes. Subsequently, polymerase chain reaction (PCR) was performed using MasterMix (ThermoScientific, Waltham, MA, USA), water, and the following primers (Sigma-Aldrich, Saint Louis, MO, USA):

**IMR1151** TGTCCAAGATGGACCAGACTCACAGGTTCTACCTGGTCTGAG

**IMR1152** ACTGGTCTGAGGCAGGGAGCATGACCAGACTCCGTCCCTCGT

The digestion process next utilizes the restriction enzyme Ddel (Promega, Madison, WI, USA) for one hour at 37°C. Following digestion, the samples were electrophoresed on a 3% agarose gel alongside a base pair standard (bp), specifically a 50bp DNA-ladder (ThermoScientific, Waltham, MA, USA), and the image is captured using a photodocumentator. Genotype determination relies on the size of the bands observed on the gel: bands of 55 and 100bp signify the mutant animal (BTBR^ob/ob^); bands of 55, 100, and 155bp indicate the heterozygous animal, while the presence of only the 155bp band denotes the wild-type BTBR animal **(Figure S1)**.


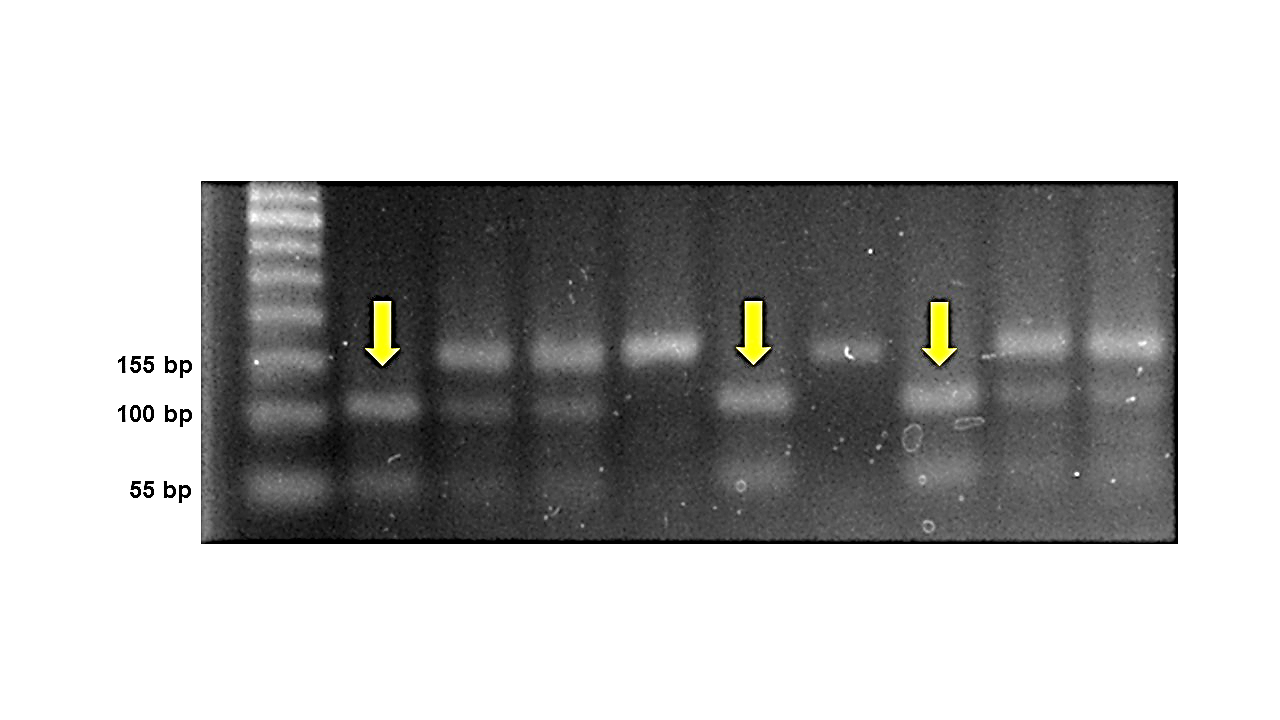


**Supplementary Figure S1**. Genotyping of BTBR^ob/ob^ animals. Arrows indicate the mutant genotype (ob/ob).

**RESULTS**

**The brown adipose tissue (BAT) and white adipose tissue (WAT) Tx group shows functional comparability to the wild-type group**

We evaluated the potential of the Tx method to reverse the diabetic and obesity phenotype by comparing functional data with BTBR^ob/ob^ and wild-type mice.

Initially, in the body weight analysis, wild-type mice demonstrated lower weight gain over time compared to both the BTBR^ob/ob^ and experimental groups, with no significant differences observed between the latter two **(Figure S2A)**. Consequently, the Tx methodology did not impact obesity in this model.

Furthermore, we investigated the Tx potential to reduce hyperglycemia. Fasting capillary blood glucose values in the Tx and BTBR wild-type groups were lower than those in the BTBR^ob/ob^ group from weeks 8 to 15. However, there were no significant differences between the Tx and wild type groups during the same time points. Notably, at 20 weeks, only the wild type group exhibited lower blood glucose levels than BTBR^ob/ob^, with no significant difference observed between the Tx and BTBR^ob/ob^ groups (**Figure S2B)**.

Additionally, we observed reduced glycosuria in Tx compared to BTBR^ob/ob^ at 9-10 and 14-15 weeks, while no significant differences were found in wild type mice compared to BTBR^ob/ob^ and Tx **(Figure S2C)**. Therefore, our results indicate that Tx mice exhibited blood glucose levels comparable to BTBR wild type, highlighting the effectiveness of this technique in reversing the diabetic phenotype.


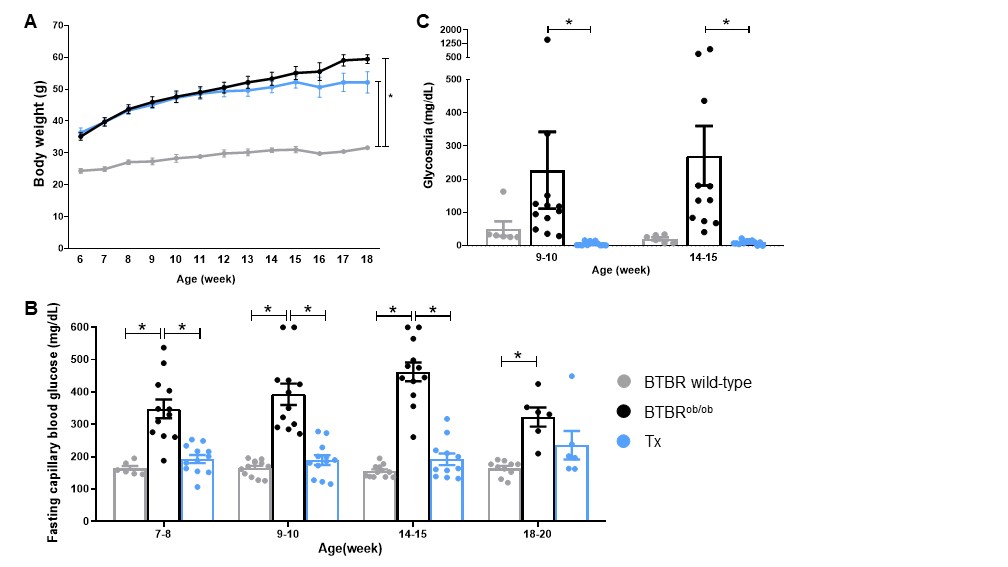


**Supplementary Figure S2**. Comparison of functional data among BTBR wild type, BTBR^ob/ob^, and Tx mice. **(A)** Variations in body weight over time. **(B)** Fasting blood glucose levels in mg/dL. **(C)** Glycosuria between groups. (*p<0.05). Error bars represent mean ± SEM; n = 6-12 animals.
